# Individual and Combined Effects of Engineered Peptides and Antibiotics on *Pseudomonas aeruginosa* Biofilms

**DOI:** 10.3390/ph10030058

**Published:** 2017-06-25

**Authors:** Biswajit Mishra, Guangshun Wang

**Affiliations:** Department of Pathology and Microbiology, College of Medicine, University of Nebraska Medical Center, 986495 Nebraska Medical Center, Omaha, NE 68198-6495, USA; Biswajit.mishra@unmc.edu

**Keywords:** antimicrobial peptides, antibiotics, biofilms, combination therapy, cathelicidin LL-37, *Pseudomonas aeruginosa*

## Abstract

*Pseudomonas aeruginosa* is involved in a variety of difficult-to-treat infections frequently due to biofilm formation. To identify useful antibiofilm strategies, this article evaluated efficacy of two newly engineered cationic antimicrobial peptides (17BIPHE2 and DASamP2), traditional antibiotics, and their combinations against biofilms at different stages. 17BIPHE2 is designed based on the 3D structure of human cathelicidin LL-37 and DASamP2 is derived from database screening. While both peptides show effects on bacterial adhesion, biofilm formation, and preformed biofilms, select antibiotics only inhibit biofilm formation, probably due to direct bacterial killing. In addition, the time dependence of biofilm formation and treatment in a static in vitro biofilm model was also studied. The initial bacterial inoculum determines the peptide concentration needed to inhibit biofilm growth. When the bacterial growth time is less than 8 h, the biomass in the wells can be dispersed by either antibiotics alone or peptides alone. However, nearly complete biofilm disruption can be achieved when both the peptide and antibiotics are applied. Our results emphasize the importance of antibiofilm peptides, early treatment using monotherapy, and the combination therapy for already formed biofilms of *P. aeruginosa*.

## 1. Introduction

*Pseudomonas aeruginosa* is an opportunistic pathogen that can cause various types of infection, including nosocomial pneumonia, wounds, ear, skin, lungs, body-implanted biomaterials like catheters, and urinary tract infections [1,2]. The situation is getting worse. On the one hand, the development of antibiotic resistance makes existing antibiotics ineffective to multidrug-resistant superbugs [3]. On the other hand, bacteria can also aggregate into communities forming a tower-like structure. Biofilm formation could block the access of traditional antibiotics to dormant bacteria or persisters, leading to the need of much higher doses (10–1000 times) for an effect [4]. Surprisingly, antibiotic treatment at a sub-lethal dose using tobramycin (an aminoglycoside), norfloxacin (a fluoroquinolone) and tetracycline could induce biofilm formation [5,6]. These problems prompted us to search for better antibiofilm amendments [7]. Current monotherapies for *P. aeruginosa* infections using colistin, tobramycin, doripenem or tigecycline have led to several concerns like rapid bacterial regrowth and emergence of resistance [8,9]. As a consequence, combination therapy is pursued for the treatment of biofilm-related infections [7,10,11]. In particular, combinations of colistin + doripenem [12,13], colistin + tobramycin [14,15], and colistin + tigecycline [16] showed promising results under various disease conditions. Colistin, which is considered the last resort for treating the *P. aeruginosa* infections, is usually included in these combined treatments. However, nephrotoxicity is a concern with the use of colistin [8,17], making the search for potent and safe alternatives necessary. 

Host defense antimicrobial peptides (AMPs) have gained much attention for the development of new peptide-based antimicrobials [7,10,18,19,20,21,22]. They are present in the six life kingdoms, ranging from bacteria to animals (http://aps.unmc.edu/AP). AMPs can rapidly kill a broad spectrum of pathogens, such as bacteria, viruses, and fungi, including antibiotic-resistant pathogens [23,24]. In addition, these peptides are also involved in signaling and immune regulation. Importantly, AMPs also possess antibiofilm activity. Many of these peptides target bacterial membranes, enabling them to kill even dormant persisters hidden in biofilms [7,25,26].

In this study, we evaluate the antibiofilm ability of two newly engineered peptides: 17BIPHE2 [27] and DASamP2 [28] (sequences in Table 1). 17BIPHE2 is designed based on the major active region (GF-17) of human cathelicidin LL-37 [27,29,30,31]. Different from the natural sequence, 17BIPHE2 contains three D-amino acids that distort the regular helical structure of GF-17 bound to bacterial membranes. The loss of a regular helical structure, however, confers the peptide with desired stability to a select set of proteases, including chymotrypsin, *S. aureus* protease V8, and fungal proteinase K. Importantly, 17BIPHE2 is effective against the ESKAPE pathogens, including *Enterococcus faecium*, *Staphylococcus aureus*, *Klebsiella pneumoniae*, *Acinetobacter baumannii*, *Pseudomonas aeruginosa*, and *Enterobacter* species [27]. DASamP2 was discovered by screening a library of representative peptides selected from the Antimicrobial Peptide Database [32,33,34]. DASamP2 also has broad spectrum antimicrobial activity [28]. Although these two peptides were obtained by different methods, they both inhibit the growth of the *P. aeruginosa* PAO1 planktonic cells (i.e., individually living bacteria) at 6.25 μM (Table 1), laying the basis for our current study of their effects on antibiotic-resistant biofilms (i.e., bacterial communities). At such a peptide concentration, these peptides would have little toxic effects on human cells as the concentrations for 50% hemolysis are at least 10 fold higher (Table 1). We also studied the combined effects of these peptides on the biofilms of laboratory and clinical strains of *P. aeruginosa* with existing antibiotics, including colistin, doripenem, tobramycin and tigecycline. In three different types of experiments, AMPs show antibiofilm effects superior to traditional antibiotics. Our results underscore the importance of early treatment as well as combinatory therapy for established *P. aeruginosa* biofilms.

## 2. Materials and Methods

### 2.1 Strains, Media and Chemicals

*Pseudomonas aeruginosa* PAO1 and four clinically isolated *P. aeruginosa* strains were used in the study. Tryptic soy broth (TSB; BD Bioscience, Sparks, MD, USA) was used as the growth media for antimicrobial and antibiofilm experiments. 17BIPHE2 and DASamP2 were synthesized on Rink resin (solid-phase) by the standard Fmoc chemistry and purified to >95% by HPLC (GeneMed, San Antonio, TX, USA). Colistin and doripenem were obtained from Sigma (St. Louis, MO, USA) while tobramycin, tigecycline, and crystal violet were purchased from Fischer Scientific (Hanover Park, IL, USA). All other chemicals were obtained from Sigma unless otherwise specified.

### 2.2. Measurement of the Minimal Inhibitory Concentration (MIC)

Antimicrobial activity was assayed by following a previously reported protocol [26,31]. In short, the *P. aeruginosa* strains, freshly inoculated in TSB from overnight culture, were allowed to reach the exponential growth phase. The cultures were then diluted to 10^6^ CFU/mL. 90 µL of this culture was added to a 96 well microplate (Costar, Corning, NY, USA) containing 10 µL of serially diluted AMPs or antibiotics solutions. After incubation overnight at 37 °C for 22 h, the absorbance of the plate was read on a CHROMATE 4300 microplate reader at 630 nm (GMI, Ramsey, MN, USA). The wells containing sterilized water instead of peptide served as the positive control while sterile media was used as the negative controls. 

### 2.3. Effects on Initial Adhesion of Bacteria

Evaluation of the effectiveness for peptides, antibiotics and their combinations were tested for inhibiting the adhesion of high density bacteria to 96 wells polystyrene microtiter plates as described [35]. Briefly, *P. aeruginosa* bacterial cells were grown overnight with OD_600_ about 1.0. One hundred and eighty µL of this culture was added to 20 µL of serially diluted 10× solution of each antimicrobial agents or combinations, and was incubated for 1 h at 37 °C to allow for attachment to plastic wells. Media was then pipetted out and the wells were carefully washed with 200 μL of normal saline three times to remove the non-adherent cells. Subsequently, 200 μL of 99% methanol was added for fixation and the plates were allowed to sit for 15 min. The plates were finally aspirated and dried. Lastly, staining was done with 200 μL of 1% crystal violet in water for 5 min. Excess stain was gently rinsed off with tap water, and plates were air-dried. Further, stains were solubilized in 200 μL of 33% glacial acetic acid followed by colorimetric measurement at 600 nm on a CHROMATE microplate reader (GMI, Ramsey, MN, USA). The amounts of biofilm growth with and without compound treatments were calculated.

### 2.4. Inhibition of Biofilm Formation

Briefly, *P. aeruginosa* cells from the overnight culture were inoculated into a fresh TSB media (1:100 dilution). 180 µL of different bacterial cultures at densities ranging from 10^5^ to 10^8^ CFU/mL were prepared and delivered to flat bottom polystyrene 96-well microtiter plates containing 20 µL of serially diluted 10× AMPs solutions. Media containing bacteria and water was treated as positive control while un-inoculated media with water served as the negative control. The plates were incubated at 37 °C for 24 h. After crystal violet staining, the plates were processed in the same manner as described in the attachment assay above.

### 2.5. Disruption of Established Biofilms

The exponential phase *P. aeruginosa* cells were diluted finally to a density of 105 CFU/mL in the TSB media. 200 µL was pipetted out into each well of the 96 well microtiter plates (polystyrene, Corning Costar, NY, USA) and incubated at 37 °C for 24 h to allow biofilm formation. Media containing bacteria and water was set up as the positive control while media was used as the negative control. Media was then gently pipetted out and the biofilms were washed with normal saline to remove nonattached cells. The biofilms were treated with a solution of 10× peptide, antibiotic, or their combination (final volume 20 µL) followed by 180 µL of fresh TSB media. The plates were incubated at 37 °C for another 24 h. Quantifications of the disruption of the biofilm by the antimicrobial agents were done using crystal violet staining by following the same method described above. This method was also employed to test the efficacy of the combinations towards four clinically isolated *P. aeruginosa* strains.

### 2.6. Immature and Mature Biofilm Disruption

Effects of the disruption of early stage and matured biofilms were evaluated using a time dependent kinetics of biofilm formation and treatment. Biofilms were allowed to establish for 4, 8, 12, 16, 20 and 24 h starting with 200 µL of 10^5^ CFU/mL in the TSB media. After treatment with the compound(s), the plates were further incubated at 37 °C for 24 h. Crystal violet staining was used for biomass quantification. 

### 2.7. Live and Dead Staining Assays of Established Biofilms by Confocal Laser Scanning Microscopy

The exponential phase *P. aeruginosa* cells were diluted to a density of 10^5^ CFU/mL in the TSB media. 2 mL of this culture was added to the cuvette (Borosilicate cover glass systems, Cat. No: 155380, Nunc, Rochester, NY, USA) and was incubated at 37 °C for 24 h to establish biofilms. Media was then gently pipetted out and cuvette chambers were washed with normal saline to remove non-attached planktonic bacterial cells. The biofilms were treated with 200 µL of 10× (125 µM) 17BIPHE2, tobramycin, or both followed by the addition of 1800 µL a fresh TSB media. The cuvettes were incubated for another 24 h at 37 °C. Control cuvettes were treated with water instead of the compound(s). Chambers were then washed with normal saline. For evaluation under confocal laser scanning microscope, the remaining biofilms were stained with 10 µL of the LIVE/DEAD kit (Invitrogen Molecular Probes, Hanover Park, IL, USA) according to the manufacturer's instructions. The samples stained with SYTO-9 (green) and propidium iodide (red) were examined under a confocal microscope (Zeiss 710, Thornwood, NY, USA) and the resulting fluorescence data were processed using the Zen 2010 software (Carl Zeiss Microcopy, Thornwood, NY, USA).

## 3. Results

### 3.1. Antimicrobial Activity against Planktonic P. aeruginosa

To get an idea on the starting treatment levels of peptides, the antimicrobial activities of 17BIPHE2 and DASamP2 were tested against the planktonic *P. aeruginosa* PAO1 laboratory strain and four clinical isolates (Table 2). Both peptides were found to exert anti-pseudomonal efficacy with minimal inhibitory concentrations (MIC) between 3.1 and 6.25 µM. Colistin inhibited the bacteria at 1.56–3.1 µM, except for *P. aeruginosa* clinical strain #1, which appeared more resistant than other strains. In the antibiotics case, doripenem was most active (MIC 0.78–1.56 µM). Tobramycin was also active at a MIC of 3.1 µM. Tigecycline was least active (MIC 12.5–25 µM). These results suggest that 17BIPHE2 and DASamP2 possess antibacterial activity against *P. aeruginosa* similar to traditional antibiotics. 

### 3.2. Influence on Initial Adhesion of P. aeruginosa

**Figure 1:**
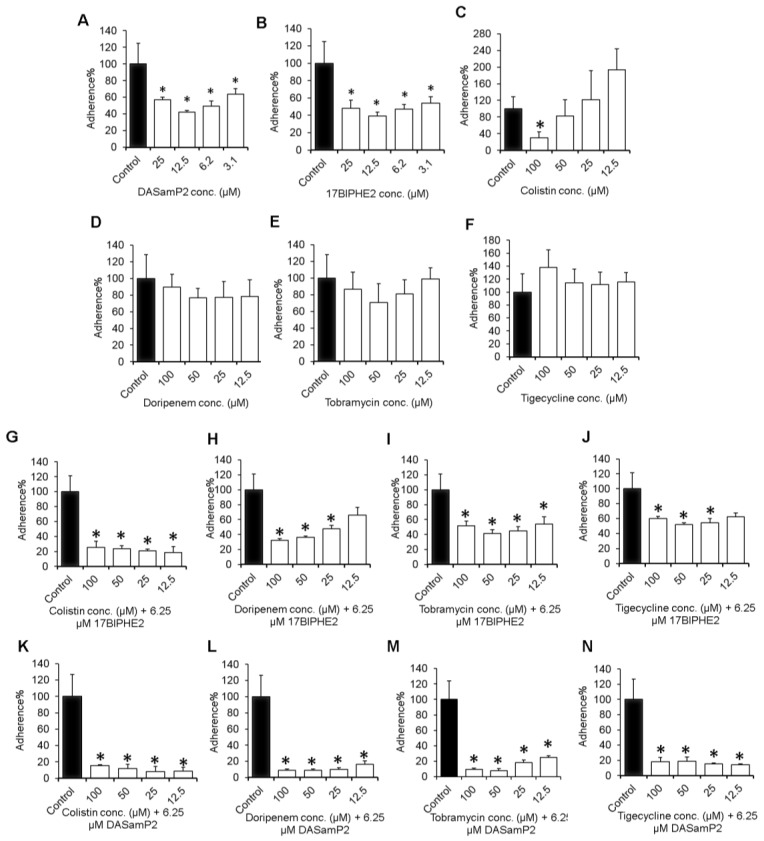
Inhibition of the attachment of *P*. *aeruginosa* PAO1 onto the polystyrene surfaces in the 96-well plate by peptides, antibiotics or their combinations. Shown are panels **A**–**C** representing peptide alone, **D**–**F** representing antibiotics alone, **G**–**J** are combination of antibiotics and 17BIPHE2 and **K**–**N** are combination of antibiotics and DASamP2, respectively. Attachment is the first step for biofilm formation. *p* values were calculated based on paired Student’s *t*-test with two tailed distribution and values <0.05 were considered significant (*).

The adhesion of bacterial cells to the polystyrene surface is considered the first step for biofilm formation. Therefore, the experiments were started from testing the ability of the individual antimicrobial agent (Table 2) in preventing the adhesion of a high bacterial inoculum OD_600_ about 1.0 onto the plastic walls of the 96-well polystyrene microtiter plates (Figure 1). At 3.1 µM, both 17BIPHE2 and DASamP2 were found to inhibit the bacterial attachment by ~50% (Figure 1A–B). It is likely that the preferred binding of cationic peptides to anionic bacteria weakened bacterial ability to adhere to the surface. In contrast, the antibiotics studied here were devoid of any anti-attachment capability (Figure 1D–F) even at a high concentration of 100 µM. However, colistin, which is a peptide antibiotic, showed a dose-dependent decrease with 70% inhibition at 100 µM (Figure 1C). To find a more effective approach, we also investigated a combined use of peptides with antibiotics. The combination effect was evident when either 17BIPHE2 (Figure 1G–J) or DASamP2 (Figure 1K–N) was applied at a dose of 6.25 µM (i.e., MIC) together with antibiotics at varying concentrations from 12.5 to 100 µM. In the case of 17BIPHE2, the best combined effect was achieved with colistin. The colistin+17BIPHE2 combination could inhibit ~80% of cells adhesion in the entire concentration range (Figure 1G), indicating that more colistin was not necessary. At the lowest concentration (12.5 µM), doripenem+17BIPHE2 only reduced the biofilm by 35%, although more was inhibited at 100 µM (Figure 1H). 

Little enhanced effects were noticed for tobramycin and tigecycline in combination with 17BIPHE2 (Figure 1I,J), and their effects were not superior to doripenem (Figure 1H). A better combined adhesion inhibitory effect was observed with DASamP2. Bacterial adhesion was impaired by these combinations (Figure 1K–N). Overall, these combination treatments led to significant reduction in the adhesion of *P. aeruginosa*.

### 3.3. Effects on Biofilm Growth of P. aeruginosa

**Figure 2:**
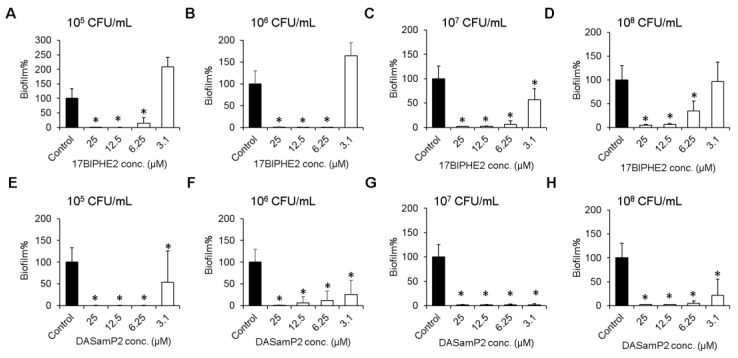
Inhibition of biofilm formation by 17BIPHE2 or DASamP2 at various amounts of *P*. *aeruginosa* PAO1 initial density: 10^5^ CFU/mL (**A** and **E**), 10^6^ CFU/mL (**B** and **F**), 10^7^ CFU/mL (**C** and **G**) and 10^8^ CFU/mL (**D** and **H**). *p* values were calculated based on paired Student’s *t*-test with two tailed distribution and values <0.05 were considered significant (*).

17BIPHE2 and DASamP2 were also found to possess an excellent biofilm inhibitory property (Figure 2). *P. aeruginosa* cells at a wide range of 10^5^–10^8^ CFU/mL were completely inhibited at a peptide concentration of 12.5 µM. However, at 6.25 µM (1× MIC) and 10^5^–10^7^ CFU/mL, both peptides nearly completely inhibited biofilm formation. Even at the relatively high bacterial density of 10^8^ CFU/mL, 60% and 70% inhibition was recorded for 17BIPHE2 and DASamP2 at their respective MIC. 

**Figure 3:**
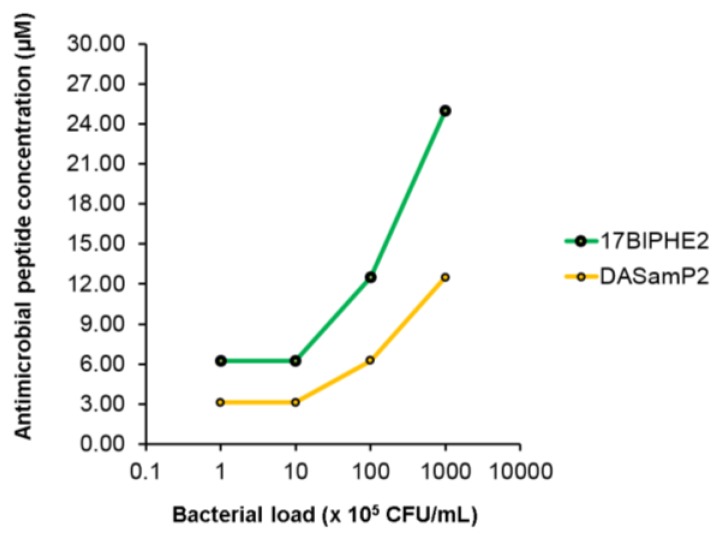
The biofilm inhibition concentration of DASamP2 or 17BIPHE2 depends on the initial bacterial density. The higher the initial bacterial load, the higher the peptide concentration needed to inhibit biofilm growth.

It is evident that the peptide concentration needed to inhibit bacterial biofilm growth depends on the initial bacterial density (Figure 3). Interestingly, the antibiotic also inhibited the biofilm growth of *P. aeruginosa* PAO1 at 10^8^ CFU/mL, except for tigecycline (Figure S1). Since the peptide alone was effective in inhibiting biofilm growth, there was no need to evaluate the combined effect of antibiotic with AMPs in this case. 

### 3.4. Impact on the Established Biofilms of P. aeruginosa

**Figure 4:**
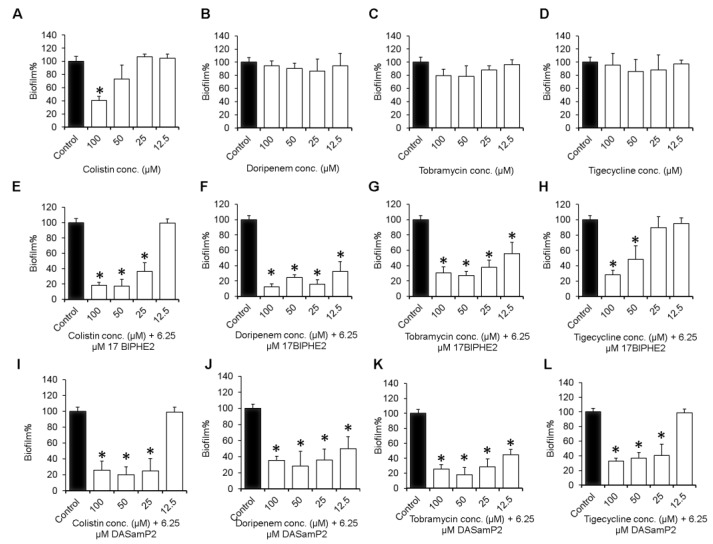
Disruption of the 24 h established biofilms of *P. aeruginosa* PAO1. Shown are panels **A**–**D** representing antibiotics alone, **E**–**H** are combination of antibiotics + 17BIPHE2 and **I**–**L** are antibiotics + DASamP2, respectively. *p* values were calculated based on paired Student’s *t*-test with two tailed distribution and values <0.05 were considered significant (*).

While the above inhibition assays deal with the early and middle stages of biofilm formation, the late stage of biofilms (i.e. preformed biofilms) could be more challenging for treatment. Therefore, we also evaluated the antibiofilm impacts of the individual compound and combinations on mature biofilms established in 24 h. Doripenem, tobramycin, tigecycline (Figure 4B–D), and DASamP2 were not effective up to 100 µM (Figure S2B). However, colistin disrupted such biofilms by 60% at 100 µM (Figure 4A), while 17BIPHE2 reduced biofilms of *P. aeruginosa* by about 50% at 50 µM (Figure S2A). 

When combined with a constant level of 6.25 µM 17BIPHE2 or DASamP2, an antibiotic dose-dependent activity was observed with a significant reduction in the biofilm biomass of *P. aeruginosa* at elevated concentrations (Figure 4). At the lowest antibiotic concentration (12.5 µM), doripenem or tobramycin reduced biofilm by 40–70% (Figure 4F,G,J,K), while colistin or tigecycline did not show any effect (Figure 4E,H,I,L), indicating doripenem or tobramycin worked better in combination with either peptide. Over 25 μM, most of the combinations displayed substantial biofilm disruption ability, except for tigecycline. For instance, the colistin+17BIPHE2 combination reduced biofilms by 65% at 25 µM and 80% at 100 µM (Figure 4E). Doripenem-17BIPHE2 eradicated about 90% of the biofilms at 100 µM. In combination with 17BIPHE2, tobramycin or tigecycline at 100 µM showed similar effectiveness of ~70% reduction. Interestingly at the same concentration of 6.25 µM (~MIC), 17BIPHE2 and DASamP2 showed a similar trend in almost all the combinations. At the lowest antibiotic concentration of 12.5 µM, either doripenem or tobramycin showed better results in combination with one of the peptides. Hence, additional efficacy validation of these combinations was conducted using clinical strains of *P. aeruginosa*.

### 3.5. Disruption of the Preformed Biofilms of P. aeruginosa Clinical Isolates

**Figure 5:**
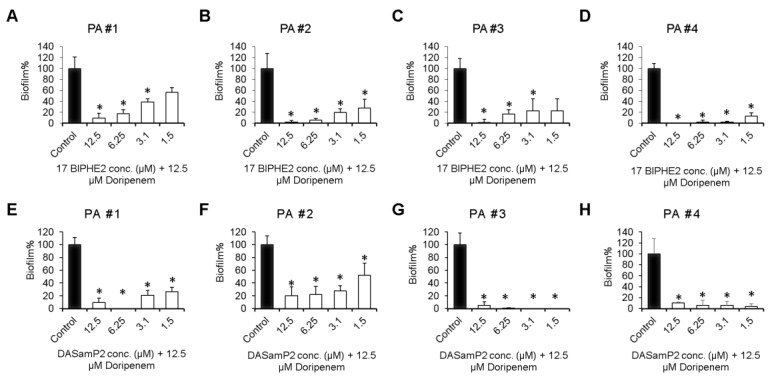
Disruption of the mature biofilms (24 h) of the *P. aeruginosa* clinical isolates with a combination of 17BIPHE2 (or DASamP2) and doripenem. Panels **A**–**D** represent combination treatment of 17BIPHE2 and doripenem against four different clinical isolates while panels **E**–**H** represent combinations between DASamP2 and doripenem against the same four clinical strains (strain #1, panels **A** and **E**; strain #2, panels **B** and **F**; strain #3, panels **C** and **G**; and strain #4, panels **D** and **H**). *p* values were calculated based on paired Student’s *t*-test with two tailed distribution and values <0.05 were considered significant (*).

Also tested was the efficacy of 17BIPHE2 or DASamP2 in combination with doripenem against the biofilms of four clinically isolated *P. aeruginosa* (Figure 5). Here the peptide concentration varied from 1.5 to 12.5 µM by keeping the doripenem concentration fixed at 12.5 µM. All the combinations were found to be very effective. For both 17BIPHE2 and DASamP2, a high dose of peptide (6.2–12.5 µM) was more effective. For example, a combination of 12.5 µM 17BIPHE2 and 12.5 µM doripenem could destroy more than 80% biofilms. There did not appear to have a clear trend regarding which peptide is better. For the clinical strains #1 (Figure 5A,E) and #3 (Figure 5C,G), DASamP2 appeared to be stronger. In the case of strain #2 (Figure 5B,F), 17BIPHE2 was better. For strain #4, both peptides behaved equally well and essentially eliminated all biofilms even at sub-MIC values (1.5–3.1 µM). Thus, in all the cases, the disruptive effects of the peptide-doripenem combination on the biofilms of these clinical strains were observed. Even a sub-MIC level of the peptide was helpful, suggesting the importance of combination treatment involving the newly designed peptides.

### 3.6. Time-dependent Biofilm Formation and Treatment Methods

**Figure 6:**
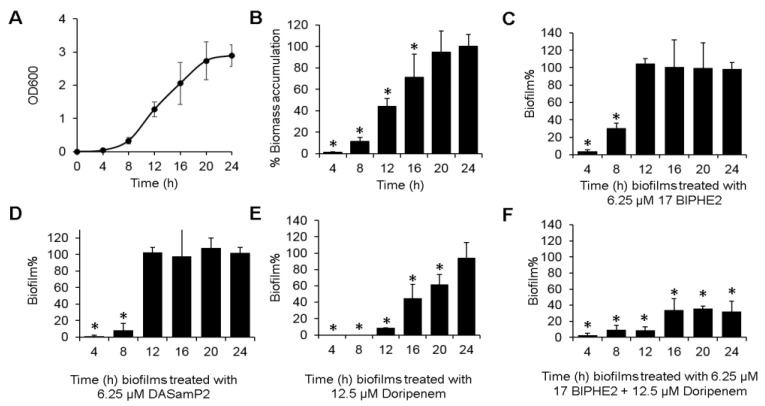
Real time biofilm formation and treatment of *P. aeruginosa* PAO1. Biofilm formation followed at every 4 h interval (**A**), time-dependent biomass accumulation in biofilms formed from 4 to 24 h (**B**), treatment of biofilms formed at each time period from 4 to 24 h by 6.25 µM 17BIPHE2 (**C**), 6.25 µM DASamP2 (**D**), 12.5 µM doripenem (**E**), and a combination of 6.25 µM 17BIPHE2 and 12.5 µM doripenem (**F**). *p* values were calculated based on paired Student’s *t*-test with two tailed distribution and values <0.05 were considered significant (*).

To provide additional insight into the biofilm treatment, we also investigated the effects of peptide alone, antibiotic alone, and their combinations on biomass formed during varying time periods (4–24 h). Biofilms of *P. aeruginosa* PAO1 were established in the same 96 well plates in the same manner. Biomass appeared to accumulate steadily with time until 24 h. This can be seen from Figure 6A as a function of the optical density (OD_600_) due to crystal violet staining, and in the plot of relative biomass change with time (assuming 100% for the 24 h matured biofilms) in Figure 6B. Interestingly, both the peptides were found to be very effective in disrupting biomass up to 8 h (6.25 µM 17BIPHE2 disrupted more than 90% of the 4 h biofilms and about 70% of the 8 h biofilms) (Figure 6C). Similarly, DASamP2 ruptured about 98% and 92% of the 4 h and 8 h biomass, respectively (Figure 6D). However, both peptides failed to disrupt the biofilms formed during 12 h or longer, indicating a critical biofilm structure already established at that time. Thus, at the early stage of biofilm progression (i.e., immature biofilms), a single administration of the peptide could be sufficient. Interestingly, doripenem alone at 12.5 µM could also do the job and it continued to reduce the biofilm mass even at 20 h (Figure 6E). However, a much better antibiofilm effect was achieved in the entire course by applying the 17BIPHE2 + doripenem combination (Figure 6F). A decrease of about 70% biofilms was obtained for 24 h matured biofilm treated with 6.25 µM 17BIPHE2 µM and 12.5 µM doripenem. These results obtained here further validated the efficacy of the same combination obtained in Figure 4F.

### 3.7. Confocal Laser Scanning Microscopy

**Figure 7:**
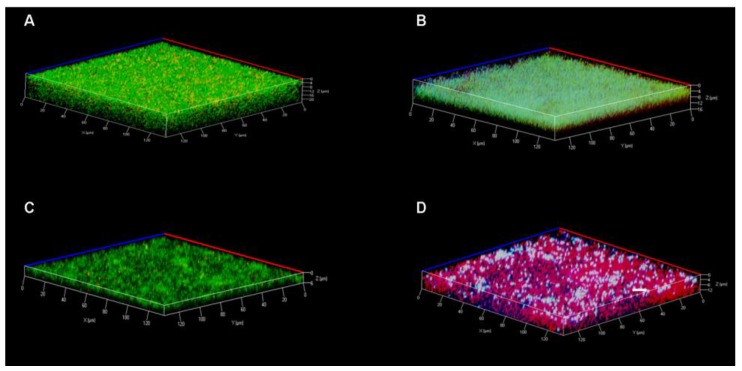
Confocal microscopic analysis of antibiofilm capability of the 17BIPHE2-tobramycin combination therapy. The *P. aeruginosa* PAO1 biofilms were stained with SYTO-9 (green for live cells) and propidium iodide (red for dead cells). In the four panels, the biofilms were treated with water in (**A**), with 12.5 µM 17BIPHE2 in (**B**), with 12.5 µM tobramycin in (**C**) and with a combination of 12.5 µM 17BIPHE2 and 12.5 µM tobramycin in (**D**). The samples were exited at 488 and 514 nm for green and red fluorescence, respectively. The color change in panel B and the white color in panel D are related to the 405 nm beam (initially thought to shine the biphenyl group of 17BIPHE2).

To provide direct evidence for bacterial death, confocal laser scanning microscopy was carried out to view the 24 h matured biofilms formed in borosilicate cuvettes treated with 17BIPHE2 alone, tobramycin alone, and a combination of both compounds (Figure 7). 

Control biofilms were treated with water only. Analysis under the microscope was done using the live and dead cell staining kit where SYTO-9 appeared green for live cells and propidium iodide (PI) appeared red for dead cells. Z-axis stacked images were taken at every 1 µm. The biofilms treated with water, 17BIPHE2, or tobramycin alone appeared mainly green in the 3D image (Figure 7A–C). However, when treated with a combination of 17BIPHE2 and tobramycin, the majority of the cells were red, indicative of dead cells (Figure 7D).

## 4. Discussion

Biofilm-related infections are more troublesome and expensive to treat [36]. It is estimated that about 80% of the chronic infections are biofilm related [37,38] and *P. aeruginosa* is one of the major causative Gram-negative pathogens for infection [39]. Currently, there are few new antibiotic candidates available [40]. Although recent treatments involving new carbapenems like doripenem (Doribax), and aminoglycosides like tobramycin were effective to treat *P. aeruginosa* infections, occurrence of carbapenem resistance [9,41] and induction of biofilms by aminoglycosides have questioned their prolonged usage [5,6]. In addition, much attention has been paid to colistin as the last resort for the treatment of complex Gram-negative infections, particularly those caused by *P. aeruginosa* [42]. However, colistin monotherapy-triggered selection pressure has now led to the emergence of colistin-resistant subpopulations [43] and its activities are also found to be compromised at high bacterial densities [44]. Therefore, this study was initiated in order to identify better candidates. 

AMPs are promising templates for developing alternative antibiotics [7,20,21,22,23,45,46,47,48]. As of April 2017, there were 2,846 natural AMPs in the regularly updated Antimicrobial Peptide Database (APD) [32,33] that constitute interesting candidates for developing novel peptide therapies. By screening the APD, we identified a potent peptide DASamP2 against *P. aeruginosa*. Compared to the original template polybia-MPI [49], DASamP2 contains additional cationic amino acids due to the following mutations: D2K, D8R and Q12R. These additional cationic amino acids might be one of the important reasons for DASamP2 to stand out in our peptide screening. Our study evaluated the potential of DASamP2 in treating the *P. aeruginosa* biofilms at various stages. Surface attachment is usually the first step for biofilm formation. DASamP2 interferes with the adhesion of *P. aeruginosa* onto the polystyrene surface (Figure 1). In addition, DASamP2 is also potent in inhibiting biofilm growth (Figure 2). These results establish the antibiofilm property of DASamP2 in vitro against *P. aeruginosa*. In addition to the antibiofilm peptide DASamP2, another database screened peptide DASamP1 also prevented the biofilm formation of *Staphylococcus aureus* USA300 in vivo [28]. Likewise, the database was successfully utilized to ab initially design the novel peptide DFTamP1, which is potent against both planktonic [50] and biofilm forms (unpublished data) of *S. aureus* USA300. Hence, the APD database is useful for identifying antibiofilm peptides.

Another peptide, 17BIPHE2, was designed based on the template of human cathelicidin LL-37, which is known to have biofilm inhibitory activity [45,47,48]. The BaAMPs database [51] registered 30 experiments for LL-37 (five on attachment, 20 on biofilm formation, and five on preformed biofilms). In the preformed biofilm cases, LL-37 alone failed to disrupt the biofilms of different bacteria, including *P. aeruginosa*. Earlier literature also revealed limited efficacy of β-lactams, carbapenems [52] and AMPs (e.g., LL-37) [53] alone in disrupting *Pseudomonas* biofilms. Shorter LL-37 fragments have also been tested [54,55]. We showed here, however, that 17BIPHE2, a 17-residue peptide engineered based on the major antimicrobial peptide (GF-17) of human LL-37 [27], is able to partially disintegrate *P. aeruginosa* mature biofilms at 8-fold the MIC, viz. 50 µM (Figure S2A). We might attribute this effect to the improved antibacterial activity of 17BIPHE2 against *P. aeruginosa* PAO1 (MIC 6.2 µM) compared to either LL-37 (MIC > 50 µM) or GF-17 (MIC 25 µM in Table S1) [29,56]. Taken together, our engineered LL-37 peptide, 17BIPHE2, possesses higher anti-pseudomonal and antibiofilm activities than either its parent molecule LL-37 or the major antimicrobial fragment GF-17. Interestingly, similar results were observed for colistin, a peptide antibiotic. In contrast, non-peptide antibiotics such as doripenem, tobramycin, and tigecycline are able to neither inhibit bacterial attachment (Figure 1) nor disperse established biofilms (Figure 4). All these results underscore the significance of cationic peptides in treating biofilms.

The antibiotic resistance issue has led to the testing of combination treatment using available antibiotics [10,11,13,25]. The colistin and carbapenem combination shows more efficient bacterial killing and less resistance tendency [57,58]. It is demonstrated that a combination of colistin with doripenem is more active than meropenem [13,58]. Likewise, colistin combined with tobramycin is more effective than individual antibiotics in decreasing bacterial biofilm in vitro as well as reducing CFU in a rat lung infection model and in patients with cystic fibrosis [15]. In addition, the same combination also decreases hospital stay and shortens the duration of antibiotic treatment in cystic fibrosis patients [59]. Another combination of colistin and tigecycline was successfully used to treat multi drug resistant *P. aeruginosa* osteomyelitis after allogeneic bone marrow transplantation [16]. To achieve a better outcome with biofilm treatment, it is natural to include other AMPs in the combination treatment for improved outcomes [60]. Indeed, human LL-37 shows a better biofilm inhibition activity when used together with antibiotics [11,61]. In this study, we tested the antibiofilm capability of DASamP2 and 17BIPHE2 against preformed biofilms of *P. aeruginosa*, which are most difficult to treat. It is of outstanding interest to note that these engineered peptides displayed antibiofilm capability in combination with existing antibiotics. Importantly, such peptide-antibiotic combinations work nicely against the biofilms of all the tested *P. aeruginosa* clinical strains as well (Figure 5). Doripenem at 12.5 µM, when combined with 1.56 µM of 17BIPHE2 or DASamP2, is sufficient to remove over 50% of the biofilms. A peptide dose-dependent activity was also observed. In fact at an equal molar concentration of peptides and the antibiotics, 80% or more of biofilms are disrupted. We should point out that the concentrations of doripenem and tobramycin used in this study are clinically relevant and such concentrations have been achieved in the blood [62,63]. Like colistin, 17BIPHE2 and DASamP2 [27,28] act on cell membranes, thereby increasing membrane permeability of existing antibiotics [13]. Our time-dependent biofilm formation studies indicate that combination treatments are essential for biofilms formed in longer than 12 h. However, combination treatment does not appear to be needed if the treatment can be applied earlier to immature biofilms (Figure 6D,E). Previously, Bowler et al. found that immature pseudomonas biofilms of less than 4 h are more susceptible to disruption by β-lactams or carbapenems [52]. We have mapped out here in detail the time dependence of biofilm treatment and found that *P. aeruginosa* immature biofilms formed in less than 8 to 12 h can be treated with either the peptide alone or doripenem alone (Figure 6). We should point out that better measurements of antibiofilm effects of compounds require a combined use of both crystal violet and XTT. While crystal violet gives the total amount of biofilms, the XTT assay measures live cells, allowing an estimation of dead bacteria in biofilms as well. We have demonstrated the advantage of this combined use in our recent study [64].

## 5. Conclusions

This study evaluated antibiofilm activity of two new peptides 17BIPHE2 and DASamP2 and their combination with traditional antibiotics against the three stages of the *P. aeruginosa* biofilms (initial attachment, middle stage growth, and mature stage) (summarized in Table 3). The results support the contention that AMPs are important templates for developing new antibiofilm peptides [7,25]. In addition, this study provides insight into biofilm treatment. The peptide concentration for inhibiting biofilm formation is dependent on initial bacterial density (Figure 3). We also found that treatment strategies are determined by the stage of the biofilms (see the paper graphics). While immature biofilms (< 8 h) can be disrupted by peptide alone or doripenem alone, mature biofilms (> 12 h) can be better destroyed by a combination of the peptide with existing antibiotics. Combination treatment involving either DASamP2 or 17BIPHE2 can better block bacterial adhesion (Figure 1). Therefore, our results also underscore the importance of early treatment to prevent the *P. aeruginosa* biofilm formation. Because 17BIPHE2 investigated here has gained stability to proteases, it could be a more promising treatment option for the preformed biofilms of *P. aeruginosa* when used in combination with tobramycin or doripenem (Figure 4).

## Figures and Tables

**Table 1:** Properties of DASamP2 and 17BIPHE2.

Peptide	DASamP2 ^a^	17BIPHE2 ^b^
Amino acid sequence	IKWKKLLRAAKRIL-NH_2_	GXKRIVQRIKDXLRNLV-NH_2_
Peptide template	Polybia-MPI	GF-17
Changes made	D2K, D8R and Q12R	F17X and F27X; X = biphenylalanine
MIC (*P. aeruginosa*) ^c^	6.25 μM	6.25 μM
HL_50_	75 μM	225 μM
Cell selectivity	12	36

^a^ Taken from ref. [28]; ^b^ Taken from ref. [27]; ^c^ MIC is the minimal inhibitory concentration; HL_50_ is the concentration that causes 50% lysis of human red blood cells; cell selectivity (or therapeutic index) is the ratio between HL_50_ and MIC.

**Table 2:** Antimicrobial activity of the selected peptides ^a^ and antibiotics against laboratory and clinical isolates of *P. aeruginosa.*

Compound	Minimal Inhibitory Concentration (µM)				
	PAO1	PA # 1	PA # 2	PA # 3	PA # 4
DASamP2	3.1–6.25	6.25	≤ 3.1	≤ 3.1	≤ 3.1
17BIPHE2	6.25	6.25	6.25	6.25	6.25
Colistin	1.56 ^b^	12.5	3.1	1.56	3.1
Doripenem	0.78–1.56	0.78	≤ 0.78	1.56	0.78
Tobramycin	3.1	3.1	≤ 3.1	≤ 3.1	≤ 3.1
Tigecycline	12.5	25	25	25	12.5

^a^ Peptides studied here include DASamP2, 17BIPHE2, and colistin; ^b^ At 1.56 μM, 80% of *P. aeruginosa* was inhibited.

**Table 3:** Impact of peptide, antibiotics and their combinations on biofilms of *P. aeruginosa* ^a^.

Biofilm Treatment Stages	Peptide Only	Antibiotic Only	Combination
Initial adhesion	√	X	√
Biofilm formation	√	√	ND
Established biofilms	√	X	√

^a^ √: Effective; X: ineffective; ND: not determined as both antibiotics and peptides are effective alone. In the established biofilm case, only 17BIPHE2 showed some disruptive effect when treated alone (see Supporting information).

## Supplementary Materials

The following are available online at http://www.mdpi.com/1424-8247/10/3/58/s1. Figure S1: Inhibition of the biofilms formation of *P. aeruginosa* PAO1 (10^8^ CFU/mL) by colistin (**A**), doripenem (**B**), tobramycin (**C**) and tigecycline (**D**). This figure indicates that, except for tigecycline, colistin, doripenem, and tobramycin could inhibit the biofilm formation of *P. aeruginosa* PAO1 on the polystyrene surfaces in the concentration range of 6.2–50 µM. *p* values were calculated based on paired Student’s *t*-test with two tailed distribution and values <0.05 were considered significant (*). Figure S2: Disruption of the 24 h preformed biofilms of *P. aeruginosa* PAO1 by 17BIPHE2 (**A**) and DASamP2 (**B**) at 12.5–100 µM. While 17BIPHE disrupted biofilms by 50–70% in this concentration range, DASamP2 only disrupted about 20% of the *P. aeruginosa* biofilms at 100 µM. It appeared that 17BIPHE2 was slightly more potent than DASamP2 when used alone. *p* values were calculated based on paired Student’s *t*-test with two tailed distribution and values <0.05 were considered significant (*). Table S1: Antimicrobial activities of the selected peptides against various pathogens
