# Supplementary material for: Individual and Combined Effects of Engineered Peptides and Antibiotics on Pseudomonas aeruginosa Biofilms
Source: Pharmaceuticals (Basel). 2017 Jun 25;10(3):58. doi: 10.3390/ph10030058 (PMC5620602; doi:10.3390/ph10030058)
Supplement: Supplementary file 1 [file pharmaceuticals-10-00058-s001.pdf]

Supplementary Materials: Individual and Combined Effects of Engineered Peptides and Antibiotics on the *Pseudomonas aeruginosa* Biofilms

Biswajit Mishra and Guangshun Wang

Table S1. Antimicrobial activities of the selected peptides against various pathogens.

Peptides	MIC (μM)				
	PA	*EC*	*KP*	*AB*	*SA*
LL-37	> 50	3.1-6.25	6.25	3.1	> 50
GF-17	25	6.25	3.1	3.1	3.1
17BIPHE2	6.25	3.1	3.1	3.1	3.1
DASamP2	3.1	≤ 3.1	≤ 3.1	≤ 1.56	≤ 3.1

PA: *Pseudomonas aeruginosa* PAO1; EC: *Escherichia coli* ATCC 25922; KP: *Klebsiella pneumoniae*; AC: *Acinetobacter baumannii*; SA: *Staphylococcus aureus* USA300.

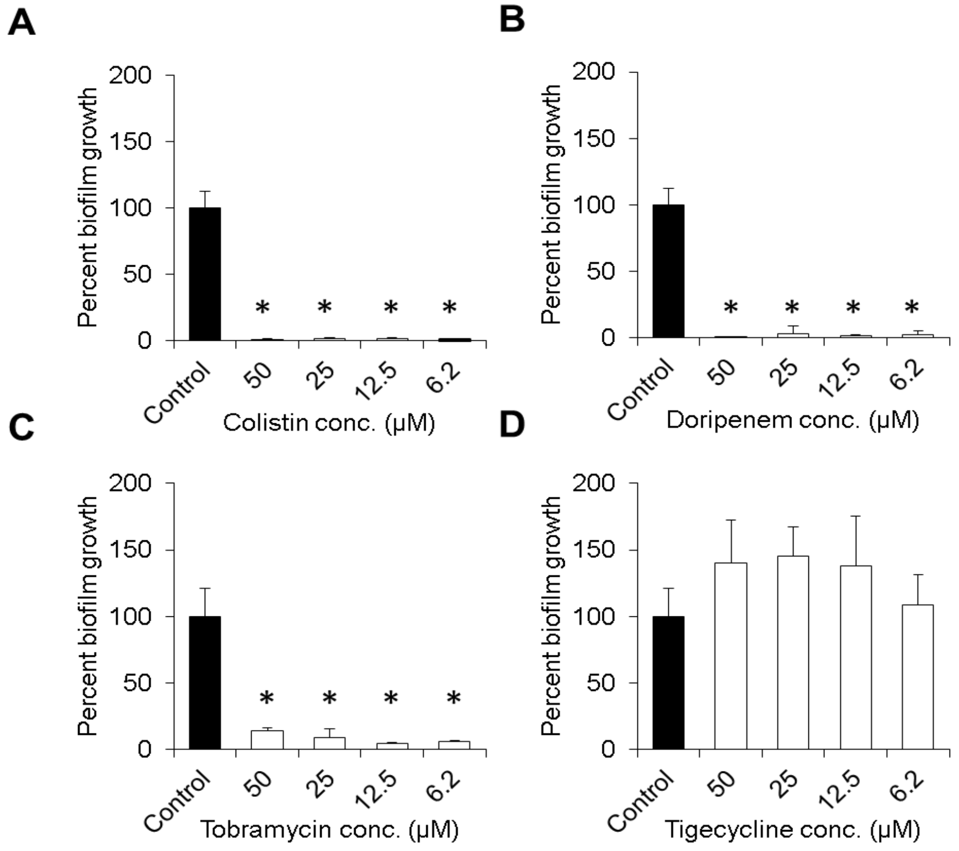

Figure S1. Inhibition of the biofilms formation of *P. aeruginosa* PAO1 (10^8 CFU/mL) by colistin (**A**), doripenem (**B**), tobramycin (**C**) and tigecycline (**D**). This figure indicates that, except for tigecycline, colistin, doripenem, and tobramycin could inhibit the biofilm formation of *P. aeruginosa* PAO1 on the polystyrene surfaces in the concentration range of 6.2–50 μM. *p* values were calculated based on paired Student's *t*-test with two tailed distribution and values <0.05 were considered significant (*).

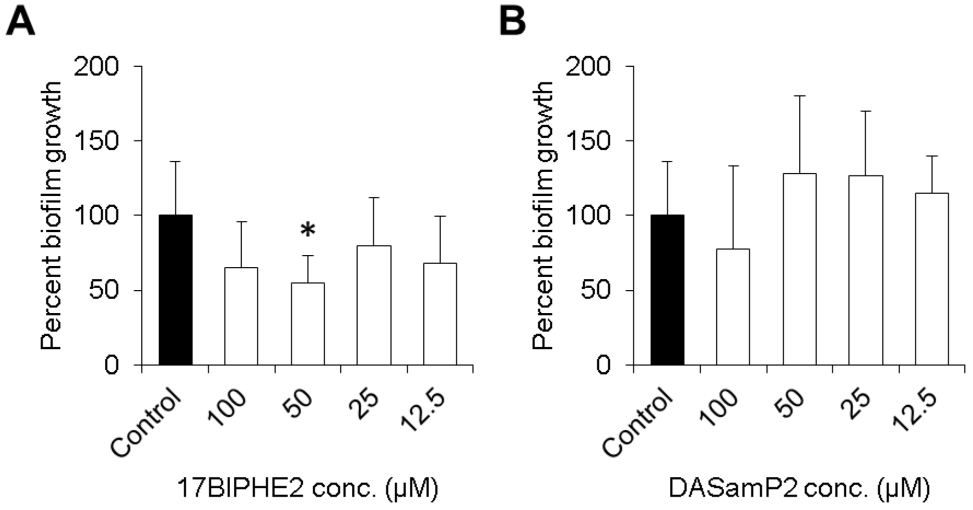

Figure S2. Disruption of the 24 h preformed biofilms of *P. aeruginosa* PAO1 by 17BIPHE2 (**A**) and DASamP2 (**B**) at 12.5–100 μM. While 17BIPHE disrupted biofilms by 50–70% in this concentration range, DASamP2 only disrupted ~20% of the *P. aeruginosa* biofilms at 100 μM. It appeared that 17BIPHE2 was slightly more potent than DASamP2 when used alone. *p* values were calculated based on paired Student's *t*-test with two tailed distribution and values <0.05 were considered significant (*).
